# Gallbladder cancer: 7-Year experience from Qatar

**DOI:** 10.1016/j.amsu.2019.06.001

**Published:** 2019-06-08

**Authors:** Ibnouf Sulieman, Walid Elmoghazy, Walid El Ansari, Ahmed Elaffandi, Hatem Khalaf

**Affiliations:** a Department of Surgery, Division of Organ Transplant, Hamad General Hospital, PO Box 3050, Doha, Qatar; b Department of Surgery, Sohag University, Sohag, Egypt; c Department of Surgery, Hamad General Hospital, Hamad Medical Corporation, PO Box 3050, Doha, Qatar; d College of Medicine, Qatar University, P.O. Box: 2713, Doha, Qatar; e School of Health and Education, University of Skövde, PO Box 408, 541 28, Skövde, Sweden; f Department of Surgical Oncology, National Cancer Institute, Cairo University, Egypt

**Keywords:** Gallbladder cancer, Epidemiology, Risk factors, Survival, Qatar, GC, Gallbladder cancer, BMI, Body Mass Index, US, Ultrasound, CT, Computed Tomography, MRI, Magnetic Resonance Imaging, PET, Positron Emission Tomography, FNAC, Fine Needle Aspiration Cytology, NET, Neuroendocrine Tumors

## Abstract

**Background:**

Gallbladder cancer (GC) is a relatively rare disease. To date, there are no studies describing the epidemiology of this disease in Qatar.

**Objective:**

To study the epidemiology of Gallbladder Cancer in Qatar.

**Methods:**

A retrospective analysis of the cases of GC in Hamad General Hospital in Qatar from 2009 to 2016.

**Results:**

Thirty-five patients presented with GC during the study period, 10 females (28.6%) and 25 males (71.4%). Fourteen patients (40%) were diagnosed incidentally after laparoscopic cholecystectomy, 16 (48.6%) were diagnosed pathologically, and 4 (11.4%) were diagnosed radiologically. The median age at diagnosis was 54 years (31–78). 74.3% of the disease occurred in patients less than 60 years old. Metastatic disease was discovered in 25 patients (71.4%) versus no metastasis in 10 patients (28.6%). The most common sites for metastasis were the liver (42.9%), peritoneum (25.7%), and lymph nodes (25.7%). Curative central hepatic resection was done in 8 patients (22.9%). Pathology showed adenocarcinoma in 27 patients (77.1%), neuroendocrine tumor in 3 patients (8.6%) and high-grade dysplasia in 1 patient (2.9%). No histopathology was available for 4 patients (11.4%). Twenty-eight patients (80.0%) had regular follow up, with 22 (62.9%) still alive. Six patients (17.1%) died during follow up with survival after diagnosis ranging from 42 days to 6.8 years.

**Conclusions:**

In Qatar, due to the unique demographics, GC is more common in males and younger age groups. Most of the patients present late with metastasis, but curative resection is associated with long-term survival.

## Highlights

• Gallbladder cancer is a relatively rare disease with a poor prognosis.
• Qatar and the other Gulf Arab countries have unique demographics, but there are no studies describing gallbladder cancer in this region.
• Gallbladder cancer in Qatar was found to be more prevalent in males and to present in younger age groups.
• Clinicians in this region should investigate gallbladder cancer more proactively in younger patients.

## 1 Introduction

Gallbladder cancer (GC) is a relatively rare disease with variable incidence worldwide [1]. Most patients are usually diagnosed after laparoscopic cholecystectomy, and many patients usually present at a late stage with poor prognosis [2]. To date, there is no data on the actual incidence and epidemiology of this disease in the State of Qatar, despite that the disease characteristics may differ in Qatar due to the unique demographic profile of the population.

Qatar is a rapidly developing country with huge development projects that led to high influxes of migrant workers and professionals from around the world [3]. The result is a multi-ethnic young adult population, with continuous and high demographic turnover, as many migrants reside for short periods before returning to their home countries. Currently, Qatar's total population is about 2.5 million, with 99% < 65 years and 1% above 65 years old. Most of these migrants are not accompanied by their families, resulting in 79% of Qatar's population being males and only 21% females [3]. Collectively, these factors reflect substantially on the epidemiology and clinicopathologic characteristics of Qatar's prevalent diseases.

The literature reveals several gaps. Given Qatar's distinctive demographic profile and population characteristics, to date, there are no studies of GC epidemiology and clinicopathologic features in Qatar, or among populations of similar demographic features in the region. Such paucity of data is a concern, considering the unique features of GC as a biliary tract malignancy with wide regional variations, rare in most western countries but much prevalent in other world regions, characterized by lack of symptoms at the early stages, and hence difficulties in treatment [4]. In addition, patients' outcomes are overall bad, surgery is the only potentially curative treatment [5], metastasis is common [6], and the lack of a serosal layer of gallbladder adjacent to the liver enables hepatic invasion/metastasis and is a major reason of its dismal prognosis [7]. These factors highlight the need for up-to-date information on the epidemiology, clinicopathological features, diagnosis and management of GC; and wide regional differences across these features mandate examining the specific characteristics of GC among our population.

Therefore, the current study assessed GC epidemiology and other features in Qatar. Our institution is the National Referral Center for all cancer patients in Qatar, receiving all GC cases diagnosed across the country. Even cases diagnosed incidentally after surgery in secondary care centers and private hospitals are eventually referred to this National Referral Center. The specific objectives of the study were to:

• Describe GC epidemiology;
• Assess potential risk factors of GC (e.g. gallstones, gallbladder polyps, BMI, environmental exposures, diabetes);
• Describe the mode of diagnosis, stage of disease at diagnosis and pattern of metastases;
• Illustrate GC histopathological types;
• Demonstrate the management modalities undertaken; and,
• Analyze the survival of GC patients and the factors associated with such survival.

Availability of accurate, up to date information on the epidemiology, prevalence, histopathology, metastasis, and management helps to assess the disease burden and guides the diagnostic and treatment decisions, while contributing to the evidence base internationally. Such information will be relevant not only to Qatar, but also to the neighboring nations in the region (e.g. United Arab Emirates, Kuwait, and Bahrain) that have similar population characteristics.

## 2 Materials and methods

### 2.1 Ethics and setting

This retrospective study was conducted at Hamad General Hospital, Doha (600-bed specialized facility). The Medical Research Centre at Hamad Medical Corporation (equivalent to the Ministry of Health) approved the study (IRB approved, Protocol #17090).

### 2.2 Registration

This study is registered with the Research Registry [8] (Research Registry UIN: research registry 4551).

### 2.3 Procedures

In this retrospective study, we systematically searched the Hepatobiliary multi-disciplinary team database at our institution and identified all GC patients over a seven-year period (July 2009–August 2016).

### 2.4 Inclusion and exclusion criteria

All adult patients (≥18 years) newly diagnosed with GC were included in the study, and the information extracted from their medical records included: demographics, risk factor/s (BMI, diabetes, gallstones, gallbladder polyps, environmental exposure), date and method of diagnosis, staging workup results, histopathological findings, treatment modality and survival time until the most recent follow-up. Patients <18 years were excluded from the study.

### 2.5 Diagnosis and staging

GC diagnosis was classified as pre-, intra- or postoperative. When patients were suspected to have GC preoperatively, the workup included abdominal ultrasound (US), Computed tomography (CT) scan, magnetic resonance imaging (MRI), with Positron emission tomography (PET/CT) for staging, and the diagnosis was confirmed by CT or US-guided core needle biopsy/fine needle aspiration cytology when feasible. When GC was suspected intraoperatively, it was confirmed by frozen section histopathology, which was the standard procedure at our hospital. Incidental postoperative diagnosis was established by formal histopathology after laparoscopic cholecystectomy. All patients were staged following the American Joint Committee on Cancer TNM staging manual (7th edition) [9].

### 2.6 Subgroup analyses

Overall and one-year survival were analyzed in relation to gender, age, the method of diagnosis, surgical intervention and presence of metastases.

### 2.7 Statistical analysis

The statistical software SPSS V. 22 (SPSS Inc., Chicago, US) was used for the analysis, with significance level set at P < 0.05. For continuous variables, the data was summarized as mean (±standard deviation) and median with range; for categorical variables, we used frequency (%). Kaplan- Meier curves were used for the calculation of patient survival.

### 2.8 Reporting

This study is reported in line with the STROCSS guidelines [10].

## 3 Results

### 3.1 Descriptive epidemiology

Table 1 shows that 35 patients (10 females, 28.6%; 25 males, 71.4%) were diagnosed with GC during the study period. The mean age at diagnosis was 52.5 (±13.5 years), and the median age was 54 years (range = 31–78 years). About 75% of the cases occurred among patients <60 years old. Patients’ ethnicities were East Asian (23 patients, 65.7%), Middle Eastern (8 patients, 22.9%), Qatari (3 patients, 8.6%), and African (one patient, 2.9%).

**Table 1:** Patient characteristics (N = 35).

Demography	Value
*Sex n (%)*	
Male	25 (71.4)
Female	10 (28.6)
*Age (years)*	
Median	54
Range	31–78
Mean ± SD	52.5 ± 13.5
*Age groups (years)*	
30-39	7 (20.0%)
40-49	8 (22.9%)
50-59	11 (31.4%)
60-69	4 (11.4%)
70-79	5 (14.3%)
*Ethnicity n (%)*	
Qatari	3 (8.6)
Middle Eastern	8 (22.8)
East Asian	23 (65.7)
African	1 (2.9)
*BMI (Mean* ± *SD)*	27.1 ± 10.5
**Pathology n (%)**	
Adenocarcinoma	27 (77.1%)
Neuroendocrine tumors	3 (8.6%)
High grade dysplasia	1 (2.9%)
No histopathology	4 (1.4%)
**Clinical n (%)**	
*Mode of Presentation*	
Incidental at laparoscopy	14 (40)
Locally advanced	7 (20)
Metastatic	14 (40)
*TNM*	
I	1 (2.9)
II	2 (5.7%)
III	3 (8.6)
IV	25 (71.4%)
Unknown	4 (11.4)
*Diagnostic mode*	
Incidental	14 (40)
Frozen section	3 (8.6)
Core/Fine needle	14 (40)
Imaging only	4 (11.4)
*Gall bladder stones* (Yes)	19 (54.3)
*Gall bladder polyp/s* (Yes)	2 (5.7)
*Metastasis at time of diagnosis* (Yes)	25 (71.4%)

### 3.2 Risk factors

Only 4 patients (11.4%) had diabetes. Mean BMI was 27.1 (±10.5) Kg/m^2^. None of the patients had a history of previous environmental exposures or contact with occupational hazardous materials. On ultrasound imaging, 19 patients (54.3%) had gallstones ranging from tiny to 3.8 cm, and two patients (5.7%) had polyps (one with multiple tiny polyps, the other with a 13 mm polyp).

### 3.3 Diagnosis

Fourteen patients (40%) were diagnosed incidentally, and another 14 (40%) were diagnosed by pathology before surgery (12 patients by core needle biopsy, 2 by fine needle aspiration cytology - FNAC). Three patients (8.6%) were suspected to have cancer intraoperatively, and the diagnosis was confirmed by intraoperative frozen section. Four patients (11.4%) presented with advanced metastatic disease on imaging, and no histopathology was undertaken.

### 3.4 Metastases

Twenty-five patients (71.4%) had metastases at the time of diagnosis, while 10 patients (28.6%) were metastasis-free. Most common sites of metastases were: liver (42.9%), peritoneum (25.7%) and regional lymph nodes (25.7%); this was followed by lungs, supra-diaphragmatic lymph nodes, spleen, bone, and retroperitoneal lymph nodes, each involved in 5.7% of the cases. One patient (2.9%) had colonic hepatic flexure metastasis, and another (2.9%) had laparoscopic port site involvement after cholecystectomy.

### 3.5 Pathology

Pathology was available for 31 patients and included: adenocarcinoma (27 patients, 77.1%), neuroendocrine tumor (3 patients, 8.6% - out of which one had pure large cell neuroendocrine tumor) and high-grade dysplasia (1 patient, 2.9%). Four patients (11.4%) had no pathology.

### 3.6 Surgical management

None of the patients diagnosed preoperatively with needle biopsy had resection due to advanced disease. Eight patients (22.9%) had curative central liver resection and lymphadenectomy. Of these, two (5.7%) proceeded to resection following intraoperative diagnosis with frozen section, while six patients (17.1%) were diagnosed incidentally after laparoscopic cholecystectomy and were re-operated. One patient had exploration laparotomy and was found to have peritoneal carcinomatosis, and only peritoneal biopsy with frozen section was done, confirming the diagnosis.

### 3.7 Survival

Complete follow data was available for 28 patients, while 7 were lost to follow up. Mean follow up was 8.6 ± 14.5 months, ranging from 0 days (one patient incidentally diagnosed after laparoscopic cholecystectomy but never returned for follow up after surgery) to 7 years (one patient who died from myocardial infarction). Of the 28 patients with complete follow up data, 22 were alive, and 6 were dead at the time of writing of this manuscript.

**Fig. 1:**
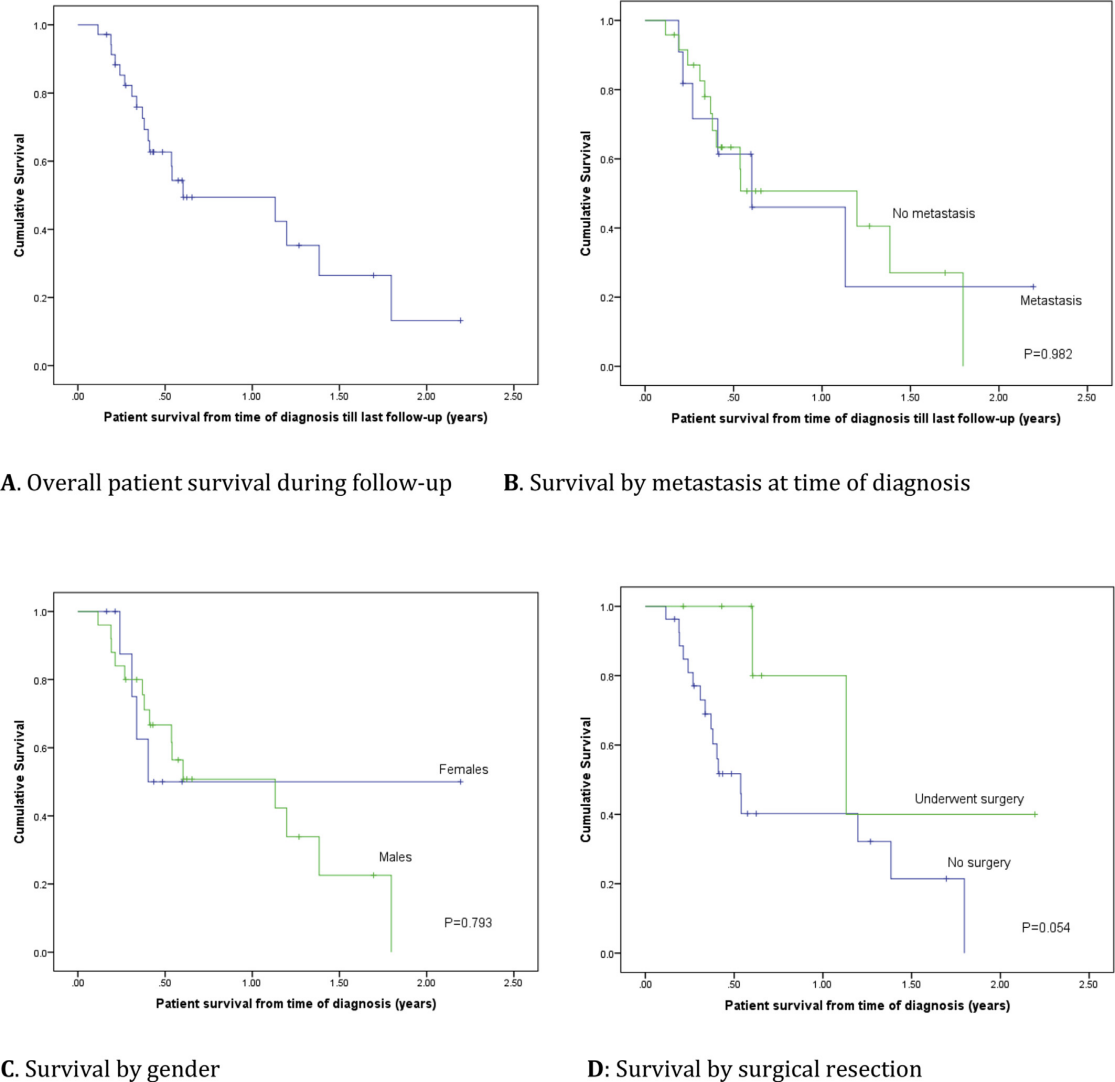
Gall bladder cancer patient survival of (n = 35): Kaplan Mayer Curves. A: Overall survival; B: Survival by metastases at time of diagnosis; C: Survival by gender; D: Survival by surgical resection.

Overall and one-year survival were analyzed in relation to gender, age (above/below 50 years), the method of diagnosis, surgical intervention and presence of metastases (Fig. 1). Overall survival was not significantly associated with any of these factors, although patients diagnosed incidentally and those who had surgical intervention tended to have better survival (94.4% *vs.* 70.6%, P = 0.58). In terms of one-year survival, those who had any surgical intervention exhibited significantly better survival than those without surgical intervention (100% *vs.* 76.5%, P = 0.02). Likewise, patients diagnosed incidentally after laparoscopic cholecystectomy had significantly better survival than those presenting with symptoms (100% *vs.* 77.8%, P = 0.02).

## 4 Discussion

GC is relatively rare, with marked variations in incidence rates, gender, ethnical and geographical distributions [1,11,12]. We diagnosed 35 GC cases in Qatar during the 7 years study period, i.e. an average of 5 cases per year, or annual incidence of 0.2/100,000, which represents the true incidence in Qatar, as our institution receives all cancer cases in the country. This is less than the worldwide incidence of 1–2.5 per 100,000, and much less than the incidence in high-risk populations like the Mapuche Indians, where it can be up to 27.3/100000 [13,14].

In terms of gender, we observed a ≈1:3 GC female/male incidence ratio, in contrast with the literature. Generally GC is more common in females, with the female/male ratio ranging between >5:1 to 1:1 (average 2–3:1) [1]. Our reversed ratio (about 1:3) mirrors the population profile in Qatar (21% females, 79% males) [3]. Although our number of GC cases among Qataris was small (3 patients), yet it comprised 2 females and 1 male, agreeing with the gender ratio seen worldwide, and also in line with that of other Middle Eastern countries (Jordan) where the ratios ranged between 3:2 and 3.7:1, and with Saudi Arabia where females comprsied 62% of the GC patients [[15], [16], [17]]. .

In terms of ethnicity, GC exhibits noticeable ethnic and geographical disparities across the world, [11,12,18]. Only one (2.9%) of our patients was African, in support of the low GC incidence in African countries, sometimes amounting to one third or one half lower than those of industrialized countries [19]. Conversely, 23 (65.7%) of our patients (20 males, 3 females) were East Asian, in agreement with research in the USA that found remarkably elevated GC incidence rates among Korean and Chinese migrants [20]. Likewise, 8 of our patients (22.9%) were from Middle Eastern countries (non-Qatari nationals), in support of others, where there was significantly higher age- and gender-standardized proportional ratios for gallbladder and other biliary cancers (1.87) among Arabic immigrant population compared with non-immigrants in the USA [21]. Others have highlighted the importance of birthplace, length of stay, and effect of migration from high- to low-risk regions in GC development and etiology, suggesting varied roles of geographic, environmental, genetic and lifestyle factors [1,[22], [23], [24]].

In terms of age (Table 1), a notable contrast between our findings and published studies [7], is the relatively younger age at diagnosis in the current study (75% were <60 years old). Although the commonest presentation of GC worldwide is between the 7th-8th decades [7], our patients presented most commonly two decades younger, and 20% were even < 40 years old at presentation. Our patients were also younger than those reported from other neighboring countries e.g. Saudi Arabia, where most patients were 66–70 years old [17]. The reasons behind our younger age patients are difficult to speculate, but demographic and ethnic factors might play a role. The key implication is that, in this region, health professionals need to have a higher index of suspicion for GC among younger patients, which calls for more proactive work-up among this age group to rule out GC. Another point is that younger GC patients have better survival, partly explained by the better performance status and better chemotherapy tolerance [25] and this again calls for more aggressive therapeutic interventions among these younger patients.

As for risk factors, in terms of cholelithiasis, 19 of our patients (54.3%) had gallstones that ranged from tiny to 3.8 cm. GC is usually associated with gallstones [11], and while some studies [26,27] reported a robust association between large gallstones (>3 cm) and GC risk; others [28] found no variation in GC risk by gallstone size.

As for gallbladder polyps, two patients (5.6%) had cholesterol polyps. Gallbladder polyps on imaging workup are concerning, as any polyp could represent GC cancer. Despite this, 95% of polyps are not neoplastic, and only 5% are neoplastic (≈4% adenomas, < 1% malignant). The progression from adenoma to carcinoma has not been established in GC cancer as it has been in other cancers (e.g. colon cancer) [1,7]. In support, none of the lesions in this cohort had associated adenomatous changes.

The mean BMI was 27.1 (±10.5) Kg/m^2^. Most studies found a strong association between obesity and GC, more established in women [29,30], while some found no association [31,32]. It is unclear whether this association could be mediated by an increased tendency among obese persons to develop gallstones [33]. Future studies could examine such relationships.

Multiple environmental exposures (Radon, heavy metals, drugs e.g. methyldopa and isoniazid), chronic bacterial infections (Salmonella, Helicobacter), parasites (Clonorchis Sinensis), and diabetes could also increase GC risk via various mechanisms, but none of our patients had such exposure/s or infections, and we did not observe an association between diabetes and GC in our sample [1,7,34,35].

In respect to the mode of diagnosis, 14 patients (40%) were diagnosed incidentally after laparoscopic cholecystectomy, supporting that GC is commonly (47%) diagnosed incidentally [2]. A total of 71.4% of our patients presented as stage IV, consistent with the literature, where most GC patients presented with metastases, e.g. 75% in Saudi Arabia [17], 47% in USA [2], and 78% in India [36].

Pathology was available for 31 patients (4 patients did not undertake gallbladder resection or core needle biopsies), where 27 (87.0%) were adenocarcinomas, 3 (9.7%) were neuroendocrine tumors (NET), and one (3.2%) had high-grade dysplasia with no invasive component. Our neuroendocrine tumors were of a higher percentage (9.7%) than others, as these tumors are rare in other studies (1.3–2% of all GC and <0.2% of all NETs [17,37,38]. One of our neuroendocrine tumors was of the pure large cell variety, which is extremely rare, with our case being the 8th case described in the literature. Nevertheless, we agree with the literature in that most adenocarcinoma cases were poorly (50%) or moderately differentiated (38.5%) [7,18,39].

In terms of survival, GC has a poor prognosis despite advances in diagnostic/therapeutic modalities [2,15,40]. Others noted that median survival (72 months) was significantly better for patients incidentally diagnosed after cholecystectomy who exhibited no evidence of disease on re-exploration, compared to those with residual disease [2,41]. We observed no associations between the age at diagnosis, patient's gender, or surgical resection on survival, but it is possible that our small sample size and heterogeneity of the patients' GC stages meant that the study might not have had sufficient power to address such questions. In our cohort, patients who underwent liver resection with intent-to-cure tended to have better survival; however, the difference was not statistically significant (Fig. 1). Among a cohort of 102 patients [6], complete surgical resection was the main variable associated with long-term survival (63.2% five-year survival *vs.* 0% in un-resected patients), and patients who presented incidentally had a higher chance of resectability and better survival than patients who presented symptomatically [6]. Our patients exhibited similar results, but these were not statistically significant.

This study has limitations. Data about the chemical composition of the stones would have contributed information about any associations between composition and GC. A larger sample size would have permitted comparisons between the different patient subgroups. Nonetheless, our sample (35 GC patients across a 7 years) is comparable to studies from Iran (37 cases over three decades) [42] and Jordan (66 cases over 19 years) [15]. Likewise, despite that the population of Saudi Arabia is about 11 times larger than Qatar, a study reported 76 patients over 7 years [17]. The current study has strengths: it is representative of the whole state of Qatar, and it bridges the lack of GC studies in the Arabian countries of the Gulf region that have similar population structures. More importantly, it generated new findings, notably the younger age at diagnosis, which is unique to this region, hence adding new insights to what is already known.

## 5 Conclusion

GC is a silent malignancy that expresses itself clinically at an advanced stage, where curative efforts are likely to be futile, and there are no current, reliable screening methods. This renders the identification of high-risk groups extremely important because raising awareness about their high risk is the way to encourage higher diagnostic efforts upon the least suspicion of the disease. Such actions may lead to earlier diagnosis and better survival. In Qatar, GC is more common in males and among younger age groups due to the unique population gender and age structures. Most patients present late with frequent metastases at diagnosis. Curative resection, when possible, presents the only hope for a better outcome.

## Funding

No funding was received for this work.

## Provenance and peer review

Not commissioned, externally peer reviewed.

## Ethical approval

The case report was approved by the Medical Research Centre of Hamad Medical Corporation (ABHATH).

Protocol ID: 17090.

## Sources of funding

This research did not receive any specific grant from funding agencies in the public, commercial, or not-for-profit sectors.

## Author contribution

Ibnouf Sulieman: Main author. Contributed to data collection, data analysis, discussion and literature review, obtaining the required ethical approvals, and the writing up of the manuscript.

Walid Elmoghazi: Surgeon: Contributed to the conception of the research idea, the data analysis, and the writing up of the manuscript. Reviewed and edited the manuscript.

Walid El Ansari: Review of the literature; writing up of the manuscript; final review of the manuscript.

Ahmed Elaffandi: Surgeon: Reviewed and edited the manuscript.

Hatem Khalaf: Surgeon: Concept of the study; Guidance and overall responsibility of the study; review and final approval of the manuscript.

## Conflicts of interest

There are no conflicts of interest to declare.

## Research registration number

Research Registry: researchregistry4551.

## Guarantor

Dr. Ibnouf Sulieman.

Dr. Walid Elmoghazy.

Dr. Hatem Khalaf.
